# Immunogenic Profiling in Mice of a HIV/AIDS Vaccine Candidate (MVA-B) Expressing Four HIV-1 Antigens and Potentiation by Specific Gene Deletions

**DOI:** 10.1371/journal.pone.0012395

**Published:** 2010-08-24

**Authors:** Juan García-Arriaza, José Luis Nájera, Carmen E. Gómez, Carlos Oscar S. Sorzano, Mariano Esteban

**Affiliations:** 1 Department of Molecular and Cellular Biology, Centro Nacional de Biotecnología, Consejo Superior de Investigaciones Científicas (CSIC), Madrid, Spain; 2 Biocomputing Unit, Centro Nacional de Biotecnología, Consejo Superior de Investigaciones Científicas (CSIC), Madrid, Spain; The University of Chicago, United States of America

## Abstract

**Background:**

The immune parameters of HIV/AIDS vaccine candidates that might be relevant in protection against HIV-1 infection are still undefined. The highly attenuated poxvirus strain MVA is one of the most promising vectors to be use as HIV-1 vaccine. We have previously described a recombinant MVA expressing HIV-1 Env, Gag, Pol and Nef antigens from clade B (referred as MVA-B), that induced HIV-1-specific immune responses in different animal models and gene signatures in human dendritic cells (DCs) with immunoregulatory function.

**Methodology/Principal Findings:**

In an effort to characterize in more detail the immunogenic profile of MVA-B and to improve its immunogenicity we have generated a new vector lacking two genes (*A41L* and *B16R*), known to counteract host immune responses by blocking the action of CC-chemokines and of interleukin 1β, respectively (referred as MVA-B ΔA41L/ΔB16R). A DNA prime/MVA boost immunization protocol was used to compare the adaptive and memory HIV-1 specific immune responses induced in mice by the parental MVA-B and by the double deletion mutant MVA-B ΔA41L/ΔB16R. Flow cytometry analysis revealed that both vectors triggered HIV-1-specific CD4^+^ and CD8^+^ T cells, with the CD8^+^ T-cell compartment responsible for >91.9% of the total HIV-1 responses in both immunization groups. However, MVA-B ΔA41L/ΔB16R enhanced the magnitude and polyfunctionality of the HIV-1-specific CD4^+^ and CD8^+^ T-cell immune responses. HIV-1-specific CD4^+^ T-cell responses were polyfunctional and preferentially Env-specific in both immunization groups. Significantly, while MVA-B induced preferentially Env-specific CD8^+^ T-cell responses, MVA-B ΔA41L/ΔB16R induced more GPN-specific CD8^+^ T-cell responses, with an enhanced polyfunctional pattern. Both vectors were capable of producing similar levels of antibodies against Env.

**Conclusions/Significance:**

These findings revealed that MVA-B and MVA-B ΔA41L/ΔB16R induced in mice robust, polyfunctional and durable T-cell responses to HIV-1 antigens, but the double deletion mutant showed enhanced magnitude and quality of HIV-1 adaptive and memory responses. Our observations are relevant in the immune evaluation of MVA-B and on improvements of MVA vectors as HIV-1 vaccines.

## Introduction

The AIDS pandemic caused by the human immunodeficiency virus (HIV-1) is spreading worldwide, with high impact and severity in human health. The number of new infections and deaths caused by AIDS disease are increasing each year, and is particularly dramatic in developing or undeveloped countries. Therefore, the finding of an effective vaccine against HIV-1 that could control the infection and disease progression should be one of the main priorities of the developed world.

An effective HIV-1 vaccine against AIDS should, in principle, stimulate both humoral and cellular immune responses to multiple viral antigens, including structural and regulatory proteins, and to induce strong, broad, polyfunctional and durable responses [1], [2], [3], [4]. Due to the difficulty in obtaining immunogens capable of inducing high titer neutralizing antibodies with broad specificities, a focus on cellular immune responses has been one of the main efforts in developing HIV-1 vaccines. In non-human primates there is a good correlation between vaccine-induced HIV-1-specific cellular immunogenicity and protection after a challenge with a pathogenic simian/human immunodeficiency virus (SHIV) [1], [3], [4], where CD8^+^ T cells play an important role in immunity to HIV-1 [4]. However, it has not been established what immunological parameters are required for protection against HIV-1 infection. This has been particularly noticeable after knowing the results of the phase III Thai clinical trial where modest protection of about 31% against HIV-1 infection was observed in vaccinees with the combination of recombinant canarypox and gp120, in spite of poor neutralizing antibodies and of reduced T-cell responses against HIV-1 [5]. Significantly, the Thai trial suggests that improved poxvirus recombinants should be considered as components of an effective HIV/AIDS vaccine.

Many vectors have been developed to improve HIV-1 specific immune responses in animal models and humans. Some of the most promising vectors are the highly attenuated vaccinia virus strains, modified vaccinia virus Ankara (MVA) and New York vaccinia virus (NYVAC) [6]. This is due to their excellent safety profile, strong immunogenicity to HIV-1 antigens in animal models, protective immune response after SHIV challenge and strong, broad, polyfunctional and durable immune responses to HIV-1 antigens in human trials ([3], [7], [8], [9], [10], for a review [11]).

We have previously described a recombinant MVA expressing codon-optimized Env as monomeric gp120 and the polyprotein Gag-Pol-Nef of HIV-1 from clade B (referred as MVA-B), that in DNA prime/MVA boost protocols induced in mice strong immune response to the HIV-1 antigens [7]. In macaques, a similar MVA construct expressing Env (gp120 from SHIV_89.6P_) and Gag-Pol-Nef (from SIV_mac239_) showed strong specific CD4^+^ and CD8^+^ T-cell immune responses with a bias for CD8^+^, and high protection after challenge with SHIV_89.6P_ [3]. Furthermore, the expression of HIV-1 antigens from MVA-B selectively induced in human dendritic cells the expression of different cellular genes that might act as regulators of immune responses to HIV-1 antigens [12]. Based on these previous results, MVA-B has recently entered a phase I clinical trial in healthy volunteers in Spain.

Although MVA recombinants are currently in clinical trials against pathogens and tumors [11] more efficient vectors that enhance the magnitude, breath, polyfunctionality and durability of the immune responses to HIV-1 antigens are desirable. The MVA genome lacks multiple genes, totaling almost 30 kb, as a result of over 570 passages in chick embryo fibroblast cells [13]; however, the vector still retains other viral genes with immunomodulatory function that block components of the host response to infection [14], [15], [16]. In fact, MVA recombinants lacking viral genes, which antagonize host specific immune responses, have been generated and some immunological benefit has been observed. Thus, deletion of certain MVA genes, such as the viral interleukin 1β binding protein (encoded by gene *B15R* in the Western Reserve (WR) strain, *B16R* in the Copenhagen strain or its equivalent gene *MVA 184R* in MVA) [17] or the gene *A41L (MVA 153L)* which encodes a secreted glycoprotein that binds some CC-chemokines [18], [19], [20], [21], resulted in viruses that when inoculated in mice showed enhanced immunogenicity against the viral vector.

In light of the need for the development of poxvirus vectors with the capacity to induce strong, broad, polyfunctional and durable immune responses to HIV-1 antigens, in this investigation we have examined in detail the immunological behaviour of the vector MVA-B and compared it with the immunogenicity elicited by a double deletion mutant in both *A41L* and *B16R* genes (referred as MVA-B ΔA41L/ΔB16R), to assess whether the MVA-B immune response to HIV-1 antigens can be improved. Our findings in mice using a DNA prime/MVA boost protocol demonstrate a strong immunogenicity profile of MVA-B and MVA-B ΔA41L/ΔB16R. Both vectors induced HIV-1-specific CD4^+^ and CD8^+^ T-cell adaptive and memory immune responses, mostly mediated by CD8^+^ T cells. However, the deletion of the two viral immunomodulatory genes *A41L*+*B16R* significantly improves the magnitude of the HIV-1-specific CD4^+^ and CD8^+^ T-cell adaptive and memory responses. HIV-1-specific CD4^+^ T-cell responses induced by both immunization groups were polyfunctional and preferentially Env-specific. Furthermore, MVA-B induced an immunodominance of Env-specific CD8^+^ T-cell responses, while MVA-B ΔA41L/ΔB16R induced preferentially GPN-specific CD8^+^ T-cell responses, with an enhanced polyfunctional pattern. Finally, both vectors triggered similar levels of antibodies against HIV-1 Env. Thus, MVA-B can improve its immunogenicity to HIV-1 antigens by the double deletion of *A41L* and *B16R* viral genes and this double mutant is an attractive candidate vector as an HIV-1 vaccine.

## Results

### Generation and *in vitro* characterization of MVA-B ΔA41L/ΔB16R

**Figure 1:**
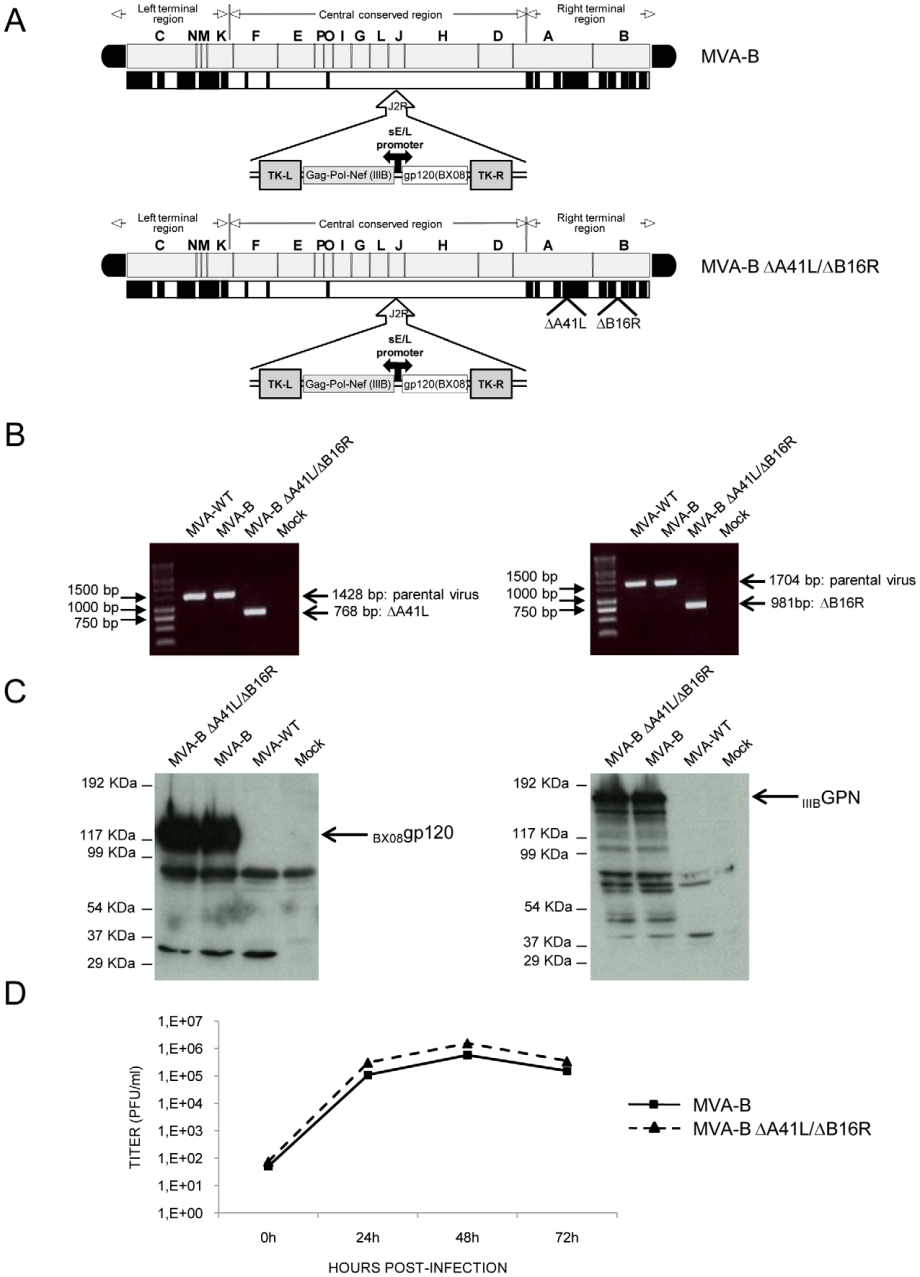
Characterization of MVA-B ΔA41L/ΔB16R recombinant virus. (A) Scheme of MVA-B and MVA-B ΔA41L/ΔB16R genome maps, adapted from [13] and [57]. The different regions are indicated by capital letters. The right and left terminal regions are shown. Below each map, the deleted or fragmented genes are depicted as black boxes. In MVA-B ΔA41L/ΔB16R the deleted *A41L* and *B16R* genes are indicated. The HIV-1 Gag-Pol-Nef (from isolate IIIB) and gp120 (from isolate BX08) clade B sequences driven by the synthetic early/late (sE/L) virus promoter inserted within the TK viral locus (J2R) are indicated, adapted from [7]. (B) PCR analysis of *A41L* and *B16R* locus. 100ng of viral DNA extracted from DF-1 cells infected at 2 PFU/cell with MVA-WT, MVA-B or MVA-B ΔA41L/ΔB16R was used for PCR analysis. The DNA products corresponding to the parental virus or to the deletion are indicated by an arrow on the right, with the expected size in base pairs. Molecular size marker (1Kb ladder) with the corresponding sizes (base pairs) is indicated on the left. Lane Mock, cells not infected. (C) Expression of HIV-1 _BX08_gp120 and _IIIB_GPN proteins in DF-1 cells infected (2 PFU/cell) with MVA-B and MVA-B ΔA41L/ΔB16R, at 24h post-infection. Arrows on the right indicate the position of HIV-1 _BX08_gp120 and _IIIB_GPN proteins. (D) Virus growth of MVA-B and MVA-B ΔA41L/ΔB16R in infected (0.01 PFU/cell) DF-1 cells at different times and titrated by plaque immunostaining assay with anti-WR antibodies. The mean of three independent experiments are shown.

An MVA-B deletion mutant lacking vaccinia virus genes *A41L* and *B16R* (termed MVA-B ΔA41L/ΔB16R), whose products act as inhibitors of CC-chemokines and IL-1β, was constructed as detailed under Materials and Methods, from the previously described recombinant MVA-B (expressing HIV-1 Env, Gag, Pol and Nef antigens from clade B) [7]. The diagram of the parental and deletion mutant is shown in Figure 1A. PCR using primers for the *A41L* and *B16R* locus confirmed the absence of these two genes in the MVA-B ΔA41L/ΔB16R genome, and their presence in MVA-B (Figure 1B). In addition, analysis by Western blot confirmed that MVA-B ΔA41L/ΔB16R expresses HIV-1 antigens _BX08_gp120 and _IIIB_GPN at the same level as their parental virus MVA-B (Figure 1C). Viral growth kinetics showed that deletion of *A41L* and *B16R* genes in the MVA-B genome does not affect virus replication and hence, these two genes are not essential for virus propagation in cultured cells (Figure 1D).

### MVA-B ΔA41L/ΔB16R enhanced the magnitude and polyfunctionality of HIV-1-specific CD4^+^ and CD8^+^ T-cell adaptive immune responses

**Figure 2:**
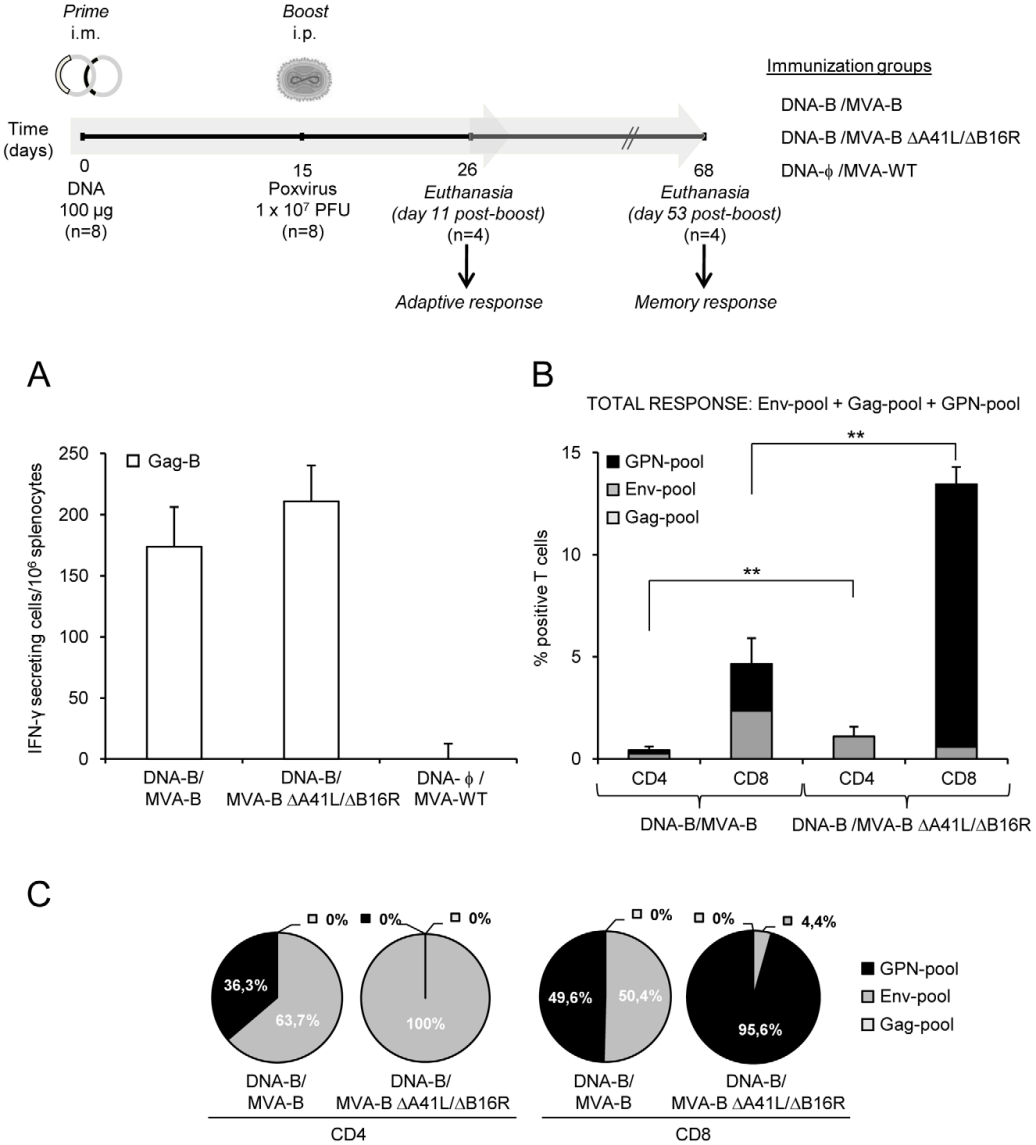
HIV-1-specific adaptive immune responses induced by MVA-B and MVA-B ΔA41L/ΔB16R. (Upper) Scheme of the DNA prime/MVA-boost immunization protocol used in this study. BALB/c mice were primed i.m with 100µg of DNA-B (50µg of pCMV-_BX08_gp120+50µg of pCDNA-_IIIB_GPN), or control DNA and two weeks later infected i.p with 1×10^7^ PFU of MVA-B, MVA-B ΔA41L/ΔB16R or MVA-WT. (A) Vaccine-elicited T-cell responses of splenocytes 11 days after the last immunization with MVA-B, MVA-B ΔA41L/ΔB16R or MVA-WT, in a fresh IFN-γ ELISPOT assay following stimulation with HIV-1 peptide Gag-B. Bars represent the total number of Gag-B-specific IFN-γ secreting cells per 10^6^ splenocytes in each group. Standard deviations from triplicate cultures are shown. *, represent statistically significant differences between groups, p<0.05. A representative experiment out of two is shown. (B) Total HIV-1-specific CD4^+^ and CD8^+^ T-cell immune responses induced in mice 11 days after the last immunization with DNA-B/MVA-B or DNA-B/MVA-B ΔA41L/ΔB16R, measured by flow cytometry using ICS assay, following stimulation with different HIV-1 peptide pools that covered the entire HIV-1 sequence present in the poxvirus vector (Env-pool, Gag-pool and GPN-pool). The percentage of HIV-1-specific CD4^+^ and CD8^+^ T-cell responses directed against Env-pool, Gag-pool and GPN-pool is indicated by different color codes, and the frequencies were calculated as the addition of single, double and triple positive T cells for the secretion of IFN-γ, TNF-α and IL-2; thus, each responding cell was counted once. The background of the unstimulated controls was subtracted in all cases, and only significant values over the background are represented. Standard deviations are shown. **, represent statistically significant differences between groups, p<0.005. (C) The pie charts summarize the data of panel B, with each set representing the fraction of CD4^+^ or CD8^+^ T cells specific for Env-pool, Gag-pool and GPN-pool.

Since DNA prime/MVA boost immunization is an effective protocol to activate T-cell responses to HIV-1 antigens [1], [2], [3], [7], [22], we analyzed the HIV-1-specific immune responses triggered in BALB/c mice by a DNA-B/MVA-B immunization regimen, and compared it with that triggered by the double deletion mutant MVA-B ΔA41L/ΔB16R. For this purpose groups of mice were first primed intramuscularly (i.m.) with 100µg of DNA-B, and two weeks later the animals were boosted by intraperitoneal (i.p.) route with 1×10^7^ PFU/mouse of recombinant viruses MVA-B or MVA-B ΔA41L/ΔB16R. Animals primed with sham DNA (DNA-φ) and boosted with the non-recombinant MVA-WT were used as control group (a diagram is shown on top of Figure 2). Vaccine-elicited adaptive immune responses in splenocytes were measured 11 days after the boost by fresh IFN-γ ELISPOT and ICS assays.

The IFN-γ ELISPOT assay, shown in Figure 2A, revealed that MVA-B ΔA41L/ΔB16R induced similar splenic T-cell responses against Gag-B (HIV-1 peptide representative of Gag antigen), in comparison with mice immunized with MVA-B. Non-recombinant MVA-WT, used as a control, did not induce HIV-1-specific responses.

To determine if immunological differences were observed between the vectors, we analyzed in more detail the phenotype of the adaptive immune response elicited by DNA-B/MVA-B and DNA-B/MVA-B ΔA41L/ΔB16R immunization groups, by polychromatic flow cytometry using ICS. To this end, we evaluated IFN-γ, TNF-α and IL-2 after *in vitro* stimulation with different HIV-1 peptide pools that covered the entire HIV-1 sequences present in the poxvirus vector (Env-pool, Gag-pool and GPN-pool). As shown in Figure 2B, at 11 days post-boost, both DNA-B/MVA-B and DNA-B/MVA-B ΔA41L/ΔB16R immunization groups induced HIV-1-specific CD4^+^ and CD8^+^ T-cell responses (determined as the sum of the individual responses obtained for Env, Gag and GPN peptide pools: Env-pool+Gag-pool+GPN-pool). The overall HIV-1-specific immune response was mainly mediated by CD8^+^ T cells (>91.9%) in both immunization groups, indicating that DNA/MVA preferentially elicited CD8^+^ T-cell responses, as previously described in macaques [3]. Furthermore, DNA-B/MVA-B ΔA41L/ΔB16R induced a significant enhancement of 2.65-fold (p<0.005) and 2.89-fold (p<0.005) in the magnitude of the total HIV-1-specific CD4^+^ and CD8^+^ T-cell responses, respectively. Significantly, some differences in the frequencies of CD4^+^ and CD8^+^ T-cell responses were observed between both groups. HIV-1-specific CD4^+^ T-cell responses were preferentially Env-specific in both immunization groups (63.7% in DNA-B/MVA-B vs. 100% in DNA-B/MVA-B ΔA41L/ΔB16R) (Figure 2C). However, while DNA-B/MVA-B induced Env-specific and GPN-specific CD8^+^ T-cell responses (50.4% and 49.6%, respectively), DNA-B/MVA-B ΔA41L/ΔB16R induced preferentially GPN-specific CD8^+^ T-cell responses (95.6%) (Figure 2C). No significant Gag-specific CD4^+^ and CD8^+^ T -cell responses were detected.

**Figure 3:**
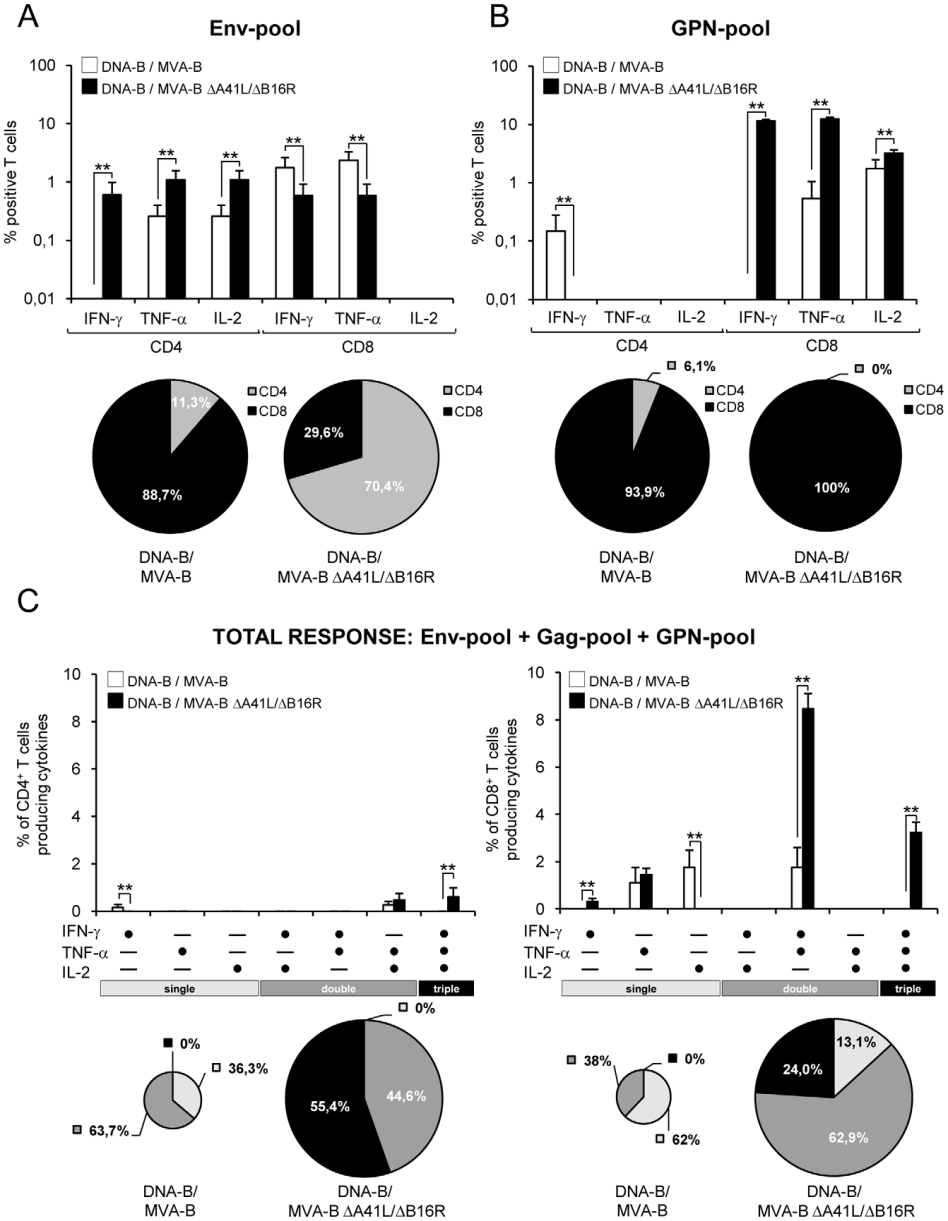
Cytokine secretion and polyfunctionality of HIV-1-specific CD4^+^ and CD8^+^ T cells after 11 days post-boost with MVA-B and MVA-B ΔA41L/ΔB16R. Mice were infected and splenocytes processed as described in Figure 2. (A and B) Percentages of CD4^+^ and CD8^+^ T cells secreting IFN-γ, TNF-α or IL-2, and directed against HIV-1 peptide pools Env-pool (A), and GPN-pool (B), and measured by ICS. The background of the unstimulated controls was subtracted in all cases, and only significant values over the background are represented. Standard deviations are shown. **, represent statistically significant differences between groups, p<0.005. Below the graphs, the percentage of CD4^+^ and CD8^+^ T cells (addition of IFN-γ+TNF-α+IL-2 CD4^+^ or CD8^+^ T-cells) specific for each HIV-1 peptide pool is depicted as pie charts. (C) Functional composition of vaccine-induced CD4^+^ and CD8^+^ T cells specific for Env-pool+Gag pool+GPN-pool, based on the secretion of IFN-γ, TNF-α and/or IL-2. All the possible combinations of the responses are shown on the X axis, whereas the percentages of the functionally distinct cell populations are shown on the Y axis. Bars correspond to the fraction of different functionally distinct T-cell population within total CD4^+^ and CD8^+^ population. Standard deviations are shown. **, represent statistically significant differences between groups, p<0.005. Responses are grouped and color-coded on the basis of the number functions. The pie chart summarizes the data and each slice of the pie correspond to the fraction of CD4^+^ or CD8^+^T cells with a given number of functions within the total CD4^+^ or CD8^+^ T-cell populations. The size of the pie chart represents the magnitude of the specific HIV-1 immune response induced.

When we analyzed the specific responses induced by the HIV-1 peptide pools, we observed that Env-specific T-cell responses in DNA-B/MVA-B were mainly induced by CD8^+^ T-cells (88.7% vs. 29.6% in DNA-B/MVA-B ΔA41L/ΔB16R; with a higher percentage of IFN-γ and TNF-α secreting T cells than DNA-B/MVA-BΔA41L/ΔB16R, p<0.005), while in DNA-B/MVA-B ΔA41L/ΔB16R the responses were mainly induced by CD4^+^ T-cells (70.4% vs. 11.3% in DNA-B/MVA-B; with a higher percentage of IFN-γ, TNF-α and IL-2 secreting T cells than DNA-B/MVA-B, p<0.005) (Figure 3A). The GPN-specific T-cell responses were mainly induced by CD8^+^ T-cells in both immunization groups (100% in DNA-B/MVA-B ΔA41L/ΔB16R vs. 93.9% in DNA-B/MVA-B), however DNA-B/MVA-B ΔA41L/ΔB16R induced a higher percentage of IFN-γ, TNF-α and IL-2 secreting T cells than DNA-B/MVA-B, (p<0.005) (Figure 3B).

The simultaneous measurements of three functions allowed the assessment of the quality of the vaccine-induced CD4^+^ and CD8^+^ T-cell responses. On the basis of the analysis of IFN-γ, TNF-α and IL-2 secretion, seven distinct HIV-1-specific CD4^+^ and CD8^+^ T-cell populations were identified. To further characterize the immunogenicity triggered in each immunized group, we assessed polyfunctional T-cell responses. The results showed that DNA-B/MVA-B ΔA41L/ΔB16R induced an enhancement in the polyfunctionality of HIV-1-specific CD4^+^ and CD8^+^ T-cell responses, with 100% of CD4^+^ T cells and 86.9% of CD8^+^ T cells secreting simultaneously 2 or 3 cytokines (Figure 3C).

The findings of Figures 2 and 3 revealed that the overall HIV-1-specific adaptive immune response triggered by MVA-B and MVA-B ΔA41L/ΔB16R was mainly mediated by CD8^+^ T-cells. However, some differences were noticeable between the vectors. Immunization with DNA-B/MVA-B ΔA41L/ΔB16R enhanced the magnitude and polyfunctionality of HIV-1-specific CD4^+^ and CD8^+^ T-cell adaptive immune responses, with an immunodominance of GPN-specific CD8^+^ T-cell responses. CD4^+^ T-cell responses induced by both vectors were Env-specific.

### MVA-B ΔA41L/ΔB16R enhanced the magnitude of long-lived memory HIV-1-specific T-cell responses, with a similar polyfunctional pattern as MVA-B

Since memory T-cell responses might be critical for protection against HIV-1 infection, we assessed at 53 days post-boost the long-term immunogenicity profile elicited by DNA-B/MVA-B and DNA-B/MVA-B ΔA41L/ΔB16R following the immunization schedule described in the top of Figure 2.

**Figure 4:**
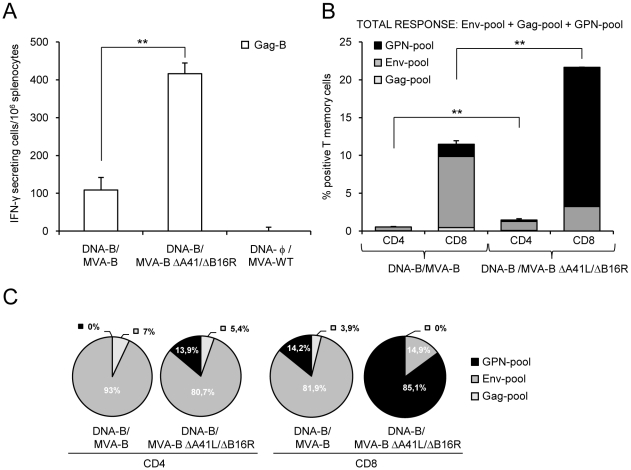
HIV-1-specific memory immune responses induced by MVA-B and MVA-B ΔA41L/ΔB16R. (A) Vaccine-elicited T-cell responses of splenocytes 53 days after the last immunization with DNA-B/MVA-B or DNA-B/MVA-B ΔA41L/ΔB16R, in a fresh IFN-γ ELISPOT assay following stimulation with HIV-1 peptide Gag-B. Bars represent the total number of Gag-B-specific IFN-γ secreting cells per 10^6^ splenocytes in each group. Standard deviations from triplicate cultures are shown. **, represent statistically significant differences between groups, p<0.005. A representative experiment out of two is shown. (B) Total HIV-1-specific CD4^+^ and CD8^+^ T-cell memory immune responses against HIV-1 peptide pools Env-pool, Gag-pool and GPN-pool, induced in mice 53 days after the last immunization with DNA-B/MVA-B or DNA-B/MVA-B ΔA41L/ΔB16R, and measured by ICS. The percentage of HIV-1-specific CD4^+^ and CD8^+^ T memory cells for Env-pool, Gag-pool and GPN-pool is indicated by different color codes and the frequencies were calculated as the addition of single and double positive T cells for the secretion of IFN-γ and IL-2; thus, each responding cell was counted once. The background of the unstimulated controls was subtracted in all cases, and only significant values over the background are represented. Standard deviations are shown. **, represent statistically significant differences between groups, p<0.005. (C) The pie charts summarize the data of panel B, with each set representing the fraction of CD4^+^ or CD8^+^ T memory cells specific for Env-pool, Gag-pool and GPN-pool. A representative experiment out of two is shown.

Vaccine-elicited memory immune responses in splenocytes were first measured by fresh IFN-γ ELISPOT assay following stimulation with HIV-1 peptide Gag-B. As shown in Figure 4A, DNA-B/MVA-B ΔA41L/ΔB16R enhanced by 3.81-fold (p<0.005) the splenic T-cell memory responses against Gag-B in comparison with mice immunized with DNA-B/MVA-B. Non-recombinant MVA-WT, used as a control, did not induce HIV-1-specific responses.

Next we decided to evaluate in more detail the phenotype of the HIV-1-specific memory T cells elicited by the immunization groups DNA-B/MVA-B ΔA41L/ΔB16R and DNA-B/MVA-B. Splenocytes were stained for CD4^+^ and CD8^+^ T cells, and we evaluated by ICS IFN-γ and IL-2 secretion after *in vitro* stimulation with the HIV-1 peptide pools for Env, Gag and GPN. Similarly as with the adaptive immune response (Figure 2B), the overall HIV-1-specific immune response at 53 days post-boost was mainly mediated by CD8^+^ T cells (>93.7%) in both immunization groups (Figure 4B). As shown in Figure 4B, long-term post-boost immunization with DNA-B/MVA-B ΔA41L/ΔB16R induced a higher magnitude of HIV-1-specific CD4^+^ and CD8^+^ T-cell memory responses producing IFN-γ and IL-2 than DNA-B/MVA-B [CD4^+^ memory T cells: 1.46% in DNA-B/MVA-B ΔA41L/ΔB16R vs. 0.56% in DNA-B/MVA-B, (p<0.005); CD8^+^ memory T cells: 21.66% in DNA-B/MVA-B ΔA41L/ΔB16R vs. 11.49% in DNA-B/MVA-B (p<0.005)]. Both vectors induced a similar pattern of HIV-1-specific CD4^+^ T-cell memory responses (with preference towards Env) (Figure 4C). However, the pattern of CD8^+^ T-cell memory responses was different between the two vectors: DNA-B/MVA-B ΔA41L/ΔB16R induced a higher percentage of GPN-specific CD8^+^ T-cell memory responses, while DNA-B/MVA-B induced preferentially a higher percentage of Env-specific CD8^+^ T-cell memory responses (Figure 4C).

**Figure 5:**
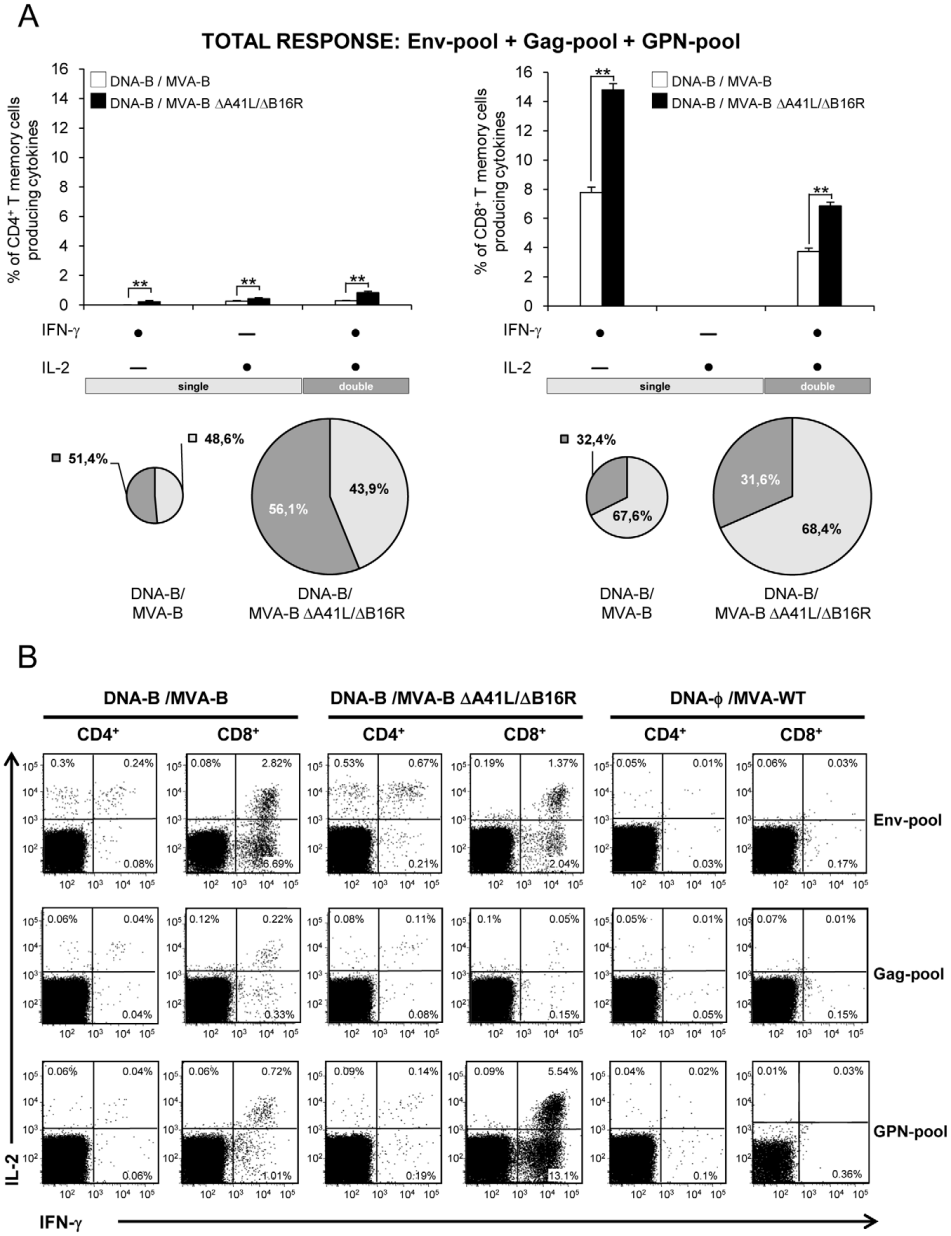
Polyfunctionality of HIV-1-specific CD4^+^ and CD8^+^ T-cell memory responses induced by immunization with MVA-B and MVA-B ΔA41L/ΔB16R. Polyfunctionality of HIV-1-specific CD4^+^ and CD8^+^ T memory cells against HIV-1 peptide pools Env-pool, Gag-pool and GPN-pool, on the basis of IFN-γ and IL-2 secretion, and induced in mice 53 days after the last immunization with DNA-B/MVA-B or DNA-B/MVA-B ΔA41L/ΔB16R, and measured by ICS assay. (A) Functional composition of vaccine-induced CD4^+^ and CD8^+^ T memory cells specific for Env-pool+Gag pool+GPN-pool, based on the secretion of IFN-γ and/or IL-2. All the possible combinations of the responses are shown on the X axis, whereas the percentages of the functionally distinct cell populations are shown on the Y axis. Bars correspond to the fraction of different functionally distinct T-cell population within total CD4^+^ and CD8^+^ population. Standard deviations are shown. **, represent statistically significant differences between groups, p<0.005. Responses are grouped and color-coded on the basis of the number functions. The pie chart summarizes the data and each slice of the pie correspond to the fraction of CD4^+^ or CD8^+^T cells with a given number of functions within the total CD4^+^ or CD8^+^ T-cell memory populations. The size of the pie chart represents the magnitude of the specific HIV-1 memory immune response induced. A representative experiment out of two is shown. (B) Representative flow cytometry plots. The numbers indicate the percentage of memory CD4^+^ or CD8^+^ T cells expressing cytokine(s) IFN-γ and/or IL-2. The last sample (CD8^+^ T memory cells GPN-specific induced after immunization with DNA-φ/MVA-WT) was lost due to contamination, and the one represented derives from another independent experiment, to show lack of response.

To have a detailed assessment of the quality of the T-cell memory responses, we evaluated secretion of two cytokines (IFN-γ and IL-2) in HIV-1-specific CD4^+^ and CD8^+^ T cells (Figure 5). In general, both immunization groups induced HIV-1-specific CD4^+^ and CD8^+^ T memory cells with a similar polyfunctional pattern consisting of cells secreting two cytokines (range between 31.6% and 56.1%). However, DNA-B/MVA-B ΔA41L/ΔB16R induced a higher magnitude of polyfunctional CD4^+^ and CD8^+^ T memory cells (Figure 5A). The percentage of polyfunctional Env-specific CD8^+^ T memory cells were higher in DNA-B/MVA-B immunization group compared with DNA-B/MVA-B ΔA41L/ΔB16R (2.82% vs. 1.37% of double CD8^+^ T memory cells that secreted IFN-γ and IL-2, p<0.005). Gag-specific T memory cells were low, thus polyfunctionality was not significant. The percentage of polyfunctional GPN-specific CD8^+^ T memory cells were higher in DNA-B/MVA-B ΔA41L/ΔB16R immunization group compared with DNA-B/MVA-B (5.54% vs. 0.72% of double CD8^+^ T memory cells that secreted IFN-γ and IL-2, p<0.005) (Figure 5B).

The findings of Figures 4 and 5 established that the overall HIV-1-specific memory immune response triggered by both vectors was mainly mediated by CD8^+^ T-cells. Immunization with DNA-B/MVA-B ΔA41L/ΔB16R significantly increased the magnitude of HIV-1-specific CD4^+^ and CD8^+^ T-cell memory responses. HIV-1-specific CD4^+^ T-cell memory responses were preferentially Env-specific in both immunization groups. However, DNA-B/MVA-B ΔA41L/ΔB16R induced an immunodominance towards CD8^+^ GPN-specific T-cell memory responses, while immunization with DNA-B/MVA-B induced preferentially CD8^+^ Env-specific T-cell memory responses. Finally, both immunization groups induced a similar polyfunctional pattern.

### MVA-B and MVA-B ΔA41L/ΔB16R induced antibodies against HIV-1 gp120

**Figure 6:**
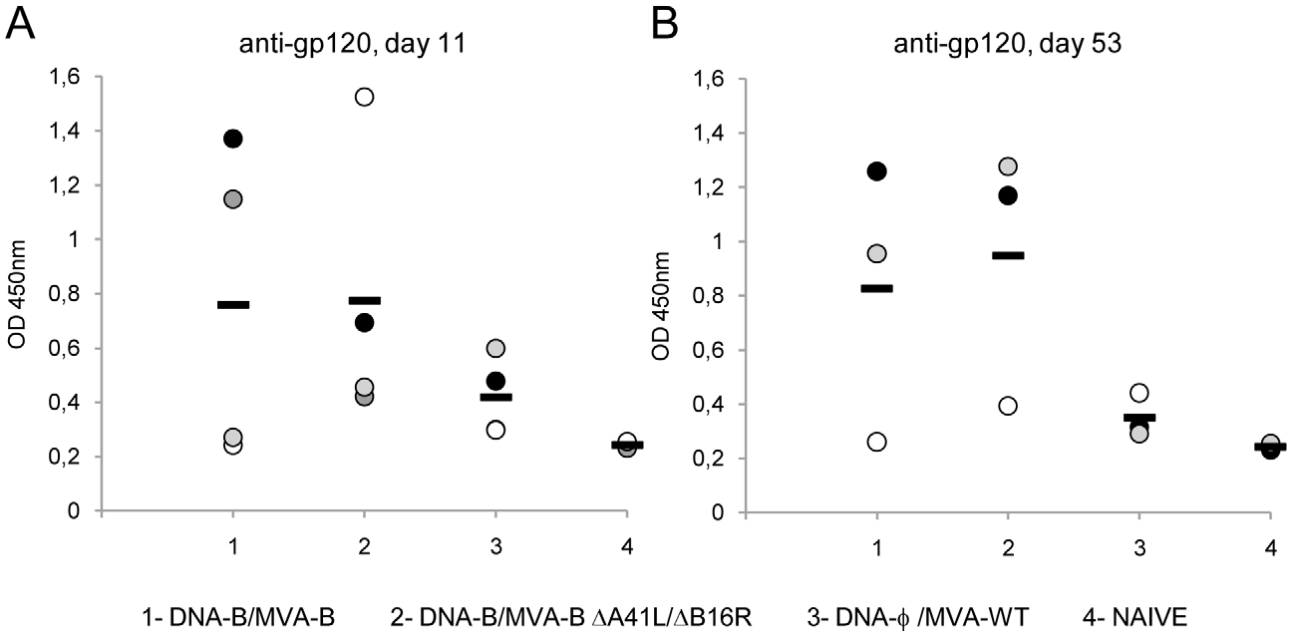
Humoral immune response elicited against HIV-1 gp160 protein induced by immunization with MVA-B and MVA-B ΔA41L/ΔB16R. Serum from individually immunized mice was evaluated by ELISA for specific anti-gp120 antibodies in blood taken 11 (A) and 53 (B) days after the last immunization with DNA-B/MVA-B, DNA-B/MVA-B ΔA41L/ΔB16R and DNA-φ/MVA-WT, as described under Materials and Methods. Serum from naïve (not immunized) animals served as control. Absorbance values (measured at 450 nm) correspond to 1/100 dilution of individual serum, and each mouse is represented by a dot. A black dash line reveals the mean value for each group. A representative experiment out of two is shown.

Since all the viral vectors release monomeric gp120 from cells in the course of virus infection [7], we also evaluated whether DNA-B/MVA-B and DNA-B/MVA-B ΔA41L/ΔB16R immunization groups elicited an antibody response against HIV-1 Env. This was performed by ELISA using individual mouse serum from each group of immunized animals at 11 and 53 days post-boost. As shown in Figure 6A and 6B, between both immunization groups, similar levels of specific antibodies reactive against gp160 protein from the HIV-1 clone LAV (clade B) were observed at the different times post-boost. Therefore, both immunization groups induced humoral immune responses against HIV-1 Env and the viral deletions did not affect the antibody levels.

## Discussion

MVA-B, the attenuated vaccinia virus vector MVA expressing the HIV-1 antigens Env, as monomeric gp120, and Gag, Pol and Nef, as a polyprotein, from clade B, is considered a vaccine candidate against HIV/AIDS [7], based on preclinical studies in different animal models [3], [7] and on gene signatures triggered in human dendritic cells [12]. In fact, expression of HIV-1 proteins from DCs infected with MVA-B induced the expression of cytokines, cytokine receptors, chemokine receptors, and molecules involved in antigen uptake and processing, including major histocompatibility complex (MHC) genes, whose products might act as regulators of immune responses to HIV-1 antigens [12]. Therefore, based on these findings, a prophylactic phase I clinical trial was initiated in Spain with MVA-B.

The MVA vector, despite of its attenuated phenotype, still contains a number of genes that encode proteins that can interfere with host immune responses [16]. It was previously described that MVA with a single deletion in *A41L* [18] or in *B16R* [17], [20] could enhance the immunogenicity of the vector. Thus, to try to improve the immunogenicity elicited by MVA-B, in this study we have removed from the viral genome these two genes (*A41L* and *B16R*), which encode proteins that interfere with the action of CC-chemokines and IL-1β, respectively and generated a double deletion mutant (MVA-B ΔA41L/ΔB16R). First, we showed in cultured cells that MVA-B ΔA41L/ΔB16R efficiently produced the four HIV-1 antigens (Env, Gag, Pol and Nef) in a stable manner and at the same level as MVA-B in the course of virus infection. Also, MVA-B ΔA41L/ΔB16R replicate similarly to MVA-B in cultured cells, indicating that deletion of these genes, has no effect on virus replication. Then, we carried out a detail characterization of the immunological responses induced in mice using DNA prime/MVA boost approach by the parental MVA-B and by the deletion mutant MVA-B ΔA41L/ΔB16R. Because flow cytometry analysis allows more extensive characterization of T-cell effector functions at the single-cell level [23], we used ICS to characterize the adaptive (11 days post-boost) and memory (53 days post-boost) HIV-1-specific immune responses induced in mice. Since our interest is to develop MVA-B mutants with enhanced immunogenicity to HIV-1 antigens, we focus our study on a detail immunological analysis by ICS on the double deletion mutant MVA-B ΔA41L/ΔB16R versus the parental MVA-B. Our findings revealed that at 11 days post-boost the magnitude and polyfunctionality of the total HIV-1-specific CD4^+^ and CD8^+^ T-cell immune responses (specific for Env-, Gag- and GPN-peptide pools) was significantly higher for MVA-B ΔA41L/ΔB16R than for MVA-B. The vaccine-induced T-cell responses were predominantly mediated by CD8^+^ T cells in both immunization groups. HIV-1-specific CD4^+^ T-cell responses were preferentially Env-specific in both immunization groups. Furthermore, immunization with DNA-B/MVA-B ΔA41L/ΔB16R induced an immunodominance towards GPN-specific CD8^+^ T-cell responses compared with DNA-B/MVA-B. At 53 days post-boost, DNA-B/MVA-B ΔA41L/ΔB16R triggered higher magnitude of CD4^+^ and CD8^+^ HIV-1-specific memory immune responses than DNA-B/MVA-B, with a significant enhancement in the percentage of CD8^+^ T memory cells that are those that have been described to have a powerful and direct antiviral capacity [24], [25], [26]. Again, as at 11 days post-boost, the vaccine-induced T-cell responses were predominantly mediated by CD8^+^ T cells, although CD4^+^ T-cell responses were also detected. Also, HIV-1-specific CD4^+^ T-cell memory responses were preferentially Env-specific in both immunization groups. DNA-B/MVA-B ΔA41L/ΔB16R induced preferentially GPN-specific CD8^+^ T memory cells whereas DNA-B/MVA-B induced an immunodominance of Env-specific CD8^+^ T memory cells. Furthermore, the memory responses induced by both immunization groups were similarly polyfunctional for the secretion of IFN-γ and IL-2.

In preclinical studies using DNA prime/poxvirus boost immunization protocols with MVA expressing the HIV-1 antigens Env, Gag, Pol and Nef, it was described an immunodominance of Env-specific responses [3], [7] This immunodominance was also reported in a phase I clinical trial with DNA/NYVAC expressing similar HIV-1 antigens but from clade C [10]. The question of immunodominance of Env has not yet been answered experimentally. The easiest explanation is that the Env protein is simply more immunogenic than Gag-Pol-Nef and when Env and the polyprotein are produced simultaneously in the infected cells (note that both Env and GPN are driven by the same synthetic early/late promoter but transcribed in opposite orientations) [7], antigen presentation is more bias for Env than GPN, probably by enhanced proteasome degradation of Env over GPN. However, the above reasoning does not apply in the DNA-B/MVA-B ΔA41L/ΔB16R immunization protocol in which a GPN response is favoured over an Env response, while both antigens are well expressed and the levels of antigen expression are similar. Thus, differences in immunodominance are more likely due to the mode of action of *A41L* and *B16R* genes playing a regulatory role in the quality of immune responses.

*A41L* encodes a secreted glycoprotein of 30 kDa [27], that binds CC-chemokines CCL21, CCL25, CCL26 and CCL28 [19], [21]. The protein A41 blocks the interaction of CC-chemokines with glycosaminoglycans on the endothelial cell surface and thereby disrupts the establishment of a chemokine concentration gradient around the site of infection. A41 is not essential for virus replication in cell culture and its deletion does not affect virus growth [27]. Deletion of *A41L* in the MVA genome enhances immunogenicity to the vector and conferred better protection against subsequent challenge with the pathogenic strain WR, thus improving vaccine efficacy [18]. The nature of the chemokines bound by A41 [19], [21] could provide an explanation for the increased immunogenicity of MVA-B ΔA41L/ΔB16R. CCL21 is a pivotal molecule for priming T-cell responses, co-stimulating the expansion of naïve CD4^+^ and CD8^+^ T cells and inducing Th1 polarization [28]. CCL25 is also involved in the formation of a T-cell response [29], and CCL28 is expressed by the mucosal epithelia of the gut, where it attracts CD4^+^ and CD8^+^ resting T cells [30]. Therefore, the deletion of *A41L* gene from MVA-B could permit to this subset of chemokines act in a way to stimulate a T-cell response against the HIV-1 antigens.

The other immunomodulatory gene, *B16R*, encodes a secreted glycoprotein of 50–60 kDa that is expressed at late times of the infectious cycle, and it functions as a viral soluble receptor for IL-1β (vIL-1βR) that blocks inflammatory and febrile host responses to infection [31], [32]. MVA expresses the B16 protein [33] and deletion of *B16R* in the MVA genome results in a virus with enhanced CD8^+^ T-cell memory responses to the virus in a mouse model [17], [20], and after vaccination MVA ΔB16R provides higher levels of protection against a challenge with WR [17]. We do not know how the absence of the viral soluble receptor for IL-1β enhanced immunogenicity of MVA-B ΔA41L/ΔB16R, although improved functionality of DCs has been proposed by the single deletion mutant of viral IL-1βR [17]. IL-1β is an essential mediator of Fas-ligation-induced maturation of murine DCs; and maturation of murine DCs could be abrogated by the use of IL-1β-neutralizing antibodies, which may function in a similar manner as the vIL1βR [34]. Thus, deletion of IL-1βR from MVA avoids the neutralization of IL-1β, and subsequently may lead to improved functionality of DCs to serve as antigen-presenting cells, which might result in better T-cell memory responses. Similarly, it has been shown that stimulation of endothelial cells with IL-1β resulted in human inducible co-stimulator-ligand-mediated activation of memory T cells [35]. The loss of neutralizing IL-1β activity might be the functional basis for our findings that vaccination with MVA-B ΔA41L/ΔB16R appeared predominantly to improve memory T-cell responses, suggesting that the viral IL-1βR could have a specific role in abrogating anti-viral memory T-cell responses.

Considering the mode of action of *A41L* and *B16R* discussed above, it might be suggested that the shift towards GPN-response triggered by the MVA double mutant is the combined result of viral-induced chemokines that stimulate T-cell responses to the HIV-1 antigens and to improved functionality of DCs to serve as antigen presenting cells. Taking into consideration that during infection gp120 is released from cells and GPN remains intracellular and that the HIV-1 proteins expressed in MVA-B-infected immature monocyte derived dendritic cells (IMDDC) induced the expression of different immunomodulatory molecules [12], it will not be surprising if the deletion of two viral genes acting as inhibitors of an inflammatory response, might have a profound effect on the shift of immune responses, with enhanced GPN over Env response. The biological significance of these findings is that enhanced Gag response has been associated with better control of virus in macaques infected with SIV and in HIV-1-infected individuals [36], [37].

It is also notorious that MVA-B ΔA41L/ΔB16R enhanced the total magnitude o the HIV-1-specific adaptive and memory immune responses, but the CD8^+^ T-cell responses elicited were mainly directed against the HIV-1 peptide pool GPN, thereby restricting the breadth of the HIV-1 immune response. While the breath of the HIV-1-specific immune response is one of the parameters that could be associated with better control of HIV-1 infection, we do not know how relevant this phenomenon is. Further experiments need to be done in other animal models as non-human primates to determine the possible benefits of the response elicited by MVA-B ΔA41L/ΔB16R.

How significant are our findings with regard to immune requirements for HIV-1 protection? While definition of correlates of protection to HIV-1 remains to be firmly established, there are a number of markers that can be used as potential indicators for an effective HIV-1 vaccine, such as: 1) specific activation of CD4^+^ and CD8^+^ T cells; 2) triggering polyfunctional responses; 3) enhanced magnitude and breath of the immune response; 4) induction of long-term memory cells; 5) production of neutralizing antibodies with broad specificities. A correlation of the CD8^+^ T-cell response with a lowering of peak viremia in acute HIV-1 infection has been described [38], [39], and there are several features of the T-cell response to HIV-1 that are correlated with control of viral replication [40], [41]. Several studies have demonstrated that polyfunctionality is associated with protective antiviral immunity [40], [41], [42], [43], [44]. In HIV-1-infected patients that are nonprogressors, HIV-1-specific CD8^+^ T cells were polyfunctional [40]. This association suggests that polyfunctional CD8^+^ T cells are an important component of a protective immune response. It might be relevant to highlight that we have obtained an immune response triggered by the two MVA vectors that fulfil several of the characteristics mentioned above for a candidate HIV-1 vaccine (i.e, activation of CD4^+^ and CD8^+^ T cells, polyfunctional response, enhanced magnitude of the immune response and induction of long-term memory cells). Moreover, we have achieved a shift and more balanced immune response to HIV-1 antigens than previously observed, by the selective deletion of the viral genes *A41L*+*B16R*.

Due to key differences in rodent and primate anatomy, physiology, immune biology [45], reproduction, and inbreeding [46], mice are considered as unreliable predictors of primate-human and nonhuman-immune responses to HIV-1 vaccine strategies (see also [47]). However, the use of mouse models has special importance as a first step to describe and characterize the impact of vectors on pathogenesis and host immune responses. In fact, the role of selected viral genes in pathogenesis when deleted from the vaccinia virus genome has been defined in mouse models, and the course of smallpox disease was first characterized in the mouse and shown similar phenotype to the human disease [48]. Moreover, the HIV-1 vaccine candidate developed in our laboratory (MVA-B) was initially tested in mice in DNA prime/MVA boost protocols showing specific immune responses to the HIV-1 antigens Env and GPN, with immunodominance for Env in both Balb/c mice and HLA-class I humanized mice [7]. A similar immune profile was observed in macaques immunized in DNA prime/MVA boost with a similar MVA vector as in mice but expressing Env (gp120 from SHIV_89.6P_) and Gag-Pol-Nef (from SIV_mac239_), revealing immunodominance for Env over GPN, strong specific CD4^+^ and CD8^+^ T-cell immune responses with a bias for CD8^+^, and high protection after challenge with SHIV_89.6_ [3]. Moreover, when a different vaccinia virus vector NYVAC was used but expressing the same cassette as MVA-B, but from subtype C, similar observations were obtained in mice [8], [49], macaques [50] and humans [10], [50], [51], with immunodominance for Env, preferential activation of CD4^+^ T cells and polyfunctional responses. Clearly these experiments and microarray data [12] demonstrated that MVA and NYVAC vectors behaved distinctly in animal and human cells, with each virus maintaining a similar profile in the different models. The promising results in mice and NHP let us to start a Phase I clinical trial with MVA-B in healthy human volunteers. Thus, detailed studies of mouse immune responses to novel HIV-1 vaccine vectors can help preclinical evaluations to optimize/compare HIV-1 immunogens or vaccine strategies for future preclinical trials in NHP and subsequently in human trials. As example, comparison of the immune response elicited by DNA prime/poxvirus (MVA or NYVAC) in preclinical (mouse and macaques) and clinical (human) trials conducted by Eurovacc is shown as supplementary information (Table S1), to indicate similarities in magnitude, breath and polyfunctional responses in the three models. Whether or not these responses are essential for the control of HIV-1 infection is not known, but complementary information on the immune response triggered in the three systems will help to define correlates of protection. The more information we obtained on specific immune responses to HIV-1 antigens and of vector impact in animal models the better chances we have to develop a more effective HIV-1 vaccine in humans.

In conclusion, this investigation has expanded our previous observations on the immunogenicity of the HIV/AIDS vaccine candidate MVA-B [7], providing a more detail characterization of the HIV-1-specific immune responses. In addition, we demonstrate that double deletion of viral genes encoding inhibitors of CC-chemokines and of IL-1β on MVA-B is an efficient approach to improve the immunogenicity of the vector against HIV-1 antigens. The immunological findings have been observed in two independent experiments indicating a consistent immunoregulatory action of the vectors. Thus, the genetic manipulation of MVA-B is an efficient strategy to expand the repertoire of T-cell immune responses against HIV-1 antigens. Since the genome of MVA contains genes encoding inhibitors of various cellular pathways and some of these inhibitors act in concert, it might be an important consideration in the improvement of the immunogenicity of MVA to knock down viral genes that act on various pathways, as we have shown here. The immunity elicited by the double mutant could be relevant in protection against HIV-1 infection, which should be explored in the macaque model. The recent excitement in the HIV-1 vaccine field as a result of the phase III Thai clinical trial [5], has pointed out in the direction that poxvirus vectors are leading vaccine candidates against HIV-1 infection and that development of modified poxvirus vectors with improved immunogenicity against HIV-1 antigens are needed. Hence, the modified MVA-B represents a promising HIV-1 vaccine candidate.

## Materials and Methods

### Ethics Statement

The animal studies were approved by the Ethical Committee of Animal Experimentation (CEEA-CNB) of Centro Nacional de Biotecnologia (CNB-CSIC) in accordance with national and international guidelines and with the Royal Decree (RD 1201/2005). Permit numbers: 152/07 and 080030.

### Cells and viruses

Primary chicken embryo fibroblast cells (CEF) [7] and DF-1 cells (a spontaneously immortalized chicken embryo fibroblast cell line. ATCC, Manassas, VA, Cat. no. CRL-12203) were grown in Dulbecco's modified Eagle's medium (DMEM) supplemented with 10% fetal calf serum (FCS). Cells were maintained in a humidified air 5% CO_2_ atmosphere at 37°C (CEF) or 39°C (DF-1). Viral infections were realized at 37°C in both cell lines. The poxvirus strains used in this work included: modified vaccinia virus Ankara (MVA) and the recombinant MVA-B expressing the HIV-1_BX08_ gp120 and HIV-1_IIIB_ Gag-Pol-Nef proteins [7]. The parental and recombinant MVA viruses were grown in CEF cells, similarly purified through two 36% (w/v) sucrose cushions, and titrated by plaque immunostaining assay as previously described [52].

### Construction of plasmid transfer vectors pGem-RG-A41L wm and pGem-RG-B16R wm

The plasmid transfer vector pGem-RG-A41L wm was used for the construction of the recombinant virus MVA-B ΔA41L/ΔB16R, with both *A41L* (*A41L* in Copenhagen strain of VACV is equivalent to *MVA 153L*) and *B16R* (*B16R* in Copenhagen strain of VACV is equivalent to *MVA 184R*) genes deleted, respectively (for simplicity, we used throughout the work the ORF nomenclature of Copenhagen strain to refer the MVA genes). pGem-RG-A41L wm was obtained by the sequential cloning of five DNA fragments containing dsRed2 and rsGFP genes and *A41L* recombination flanking sequences into the plasmid pGem-7Zf(-) (Promega). The dsRed2 gene under the control of the synthetic early/late (E/L) promoter was amplified by PCR from plasmid pG-dsRed2 with oligonucleotides Red2-B (5′-GAACTAGGATCCTAACTCGAGAAA-3′) (BamHI site underlined) and Red2-N (5′-ATTAGTATGCATTTATTTATTTAGG-3′) (NsiI site underlined) (785 bp), digested with BamHI and NsiI and inserted into the BamHI/NsiI-digested pGem-7Zf(-) to generate pGem-Red wm (3740 bp). The rsGFP gene under the control of the synthetic E/L promoter was amplified by PCR from plasmid pG-dsRed2 with oligonucleotides GFP-X (5′-CGTTGGTCTAGAGAGAAAAATTG-3′) (XbaI site underlined) and GFP-E (5′-CTATAGAATTCTCAAGCTATGC-3′) (EcoRI site underlined) (832 bp), digested with XbaI and EcoRI and inserted into plasmid pGem-Red wm previously digested with XbaI and EcoRI to obtain pGem-Red-GFP wm (4540 bp). MVA-B genome was used as the template to amplify the right flank of *A41L* gene (389 bp) with oligonucleotides RFA41L-AatII-F (5′-CCTACTGACGTCATAAGCTATAATA-3′) (AatII site underlined) and RFA41L-XbaI-R (5′-GATAATTCTAGATTGTTATTTTTAT-3′) (XbaI site underlined). This right flank was digested with AatII and XbaI and cloned into plasmid pGem-Red-GFP wm previously digested with the same restriction enzymes to generate pGem-RG-RFsA41L wm (4896 bp). The repeated right flank of *A41L* gene (389 bp) was amplified by PCR from MVA-B genome with oligonucleotides RF′A41L-EcoRI-F (5′-CCTACTGAATTCATAAGCTATAATA-3′) (EcoRI site underlined) and RF′A41L-ClaI-R (5′-GATAATATCGATTTGTTATTTTTAT-3′) (ClaI site underlined), digested with EcoRI and ClaI and inserted into the EcoRI/ClaI-digested pGem-RG-RFsA41L wm to generate pGem-RG-RFdA41L wm (5244 bp). The left flank of *A41L* gene (404 bp) was amplified by PCR from MVA-B genome with oligonucleotides LFA41L-ClaI-F (5′-TAACGAATCGATTCTGCAATATTG-3′) (ClaI site underlined) and LFA41L-BamHI-R (5′-GTGTTCGGATCCATTAGAGAGTTAG-3′) (BamHI site underlined), digested with ClaI and BamHI and inserted into the ClaI/BamHI-digested pGem-RG-RFdA41L wm. The resulting plasmid pGem-RG-A41L wm (5618 bp) was confirmed by DNA sequence analysis and directs the deletion of *A41L* gene from MVA-B genome.

The plasmid transfer vector pGem-RG-B16R wm was used for the construction of the recombinant virus MVA-B ΔA41L/ΔB16R, with both *A41L* and *B16R* genes deleted, respectively. pGem-RG-B16R wm was obtained by the sequential cloning of *B16R* recombination flanking sequences into the plasmid pGem-Red-GFP wm (previously described). MVA-B genome was used as the template to amplify the left flank of *B16R* gene (361 bp) with oligonucleotides LFB16R-AatII-F (5′-CTTTTAGACGTCATGCGGAATTAGTG-3′) (AatII site underlined) and LFB16R-XbaI-R (5′-TAGTATTCTAGATTTATTTTATAGTG-3′) (XbaI site underlined). This left flank was digested with AatII and XbaI and cloned into plasmid pGem-Red-GFP wm previously digested with the same restriction enzymes to generate pGem-RG-LFsB16R wm (4868 bp). The repeated left flank of *B16R* gene (361 bp) was amplified by PCR from MVA-B genome with oligonucleotides LF′B16R-EcoRI-F (5′-CTTTTAGAATTCATGCGGAATTAGTG-3′) (EcoRI site underlined) and LF′B16R-ClaI-R (5′-TAGTATATCGATTTTATTTTATAGTG-3′) (ClaI site underlined), digested with EcoRI and ClaI and inserted into the EcoRI/ClaI-digested pGem-RG-LFsB16R wm to generate pGem-RG-LFdB16R wm (5188 bp). The right flank of *B16R* gene (386 bp) was amplified by PCR from MVA-B genome with oligonucleotides RFB16R-ClaI-F (5′-AGTATAATCGATATGTATGTTGTTAC-3′) (ClaI site underlined) and RFB16R-BamHI-R (5′-TGTATCGGATCCCACCCTTTCCTAT-3′) (BamHI site underlined), digested with ClaI and BamHI and inserted into the ClaI/BamHI-digested pGem-RG-LFdB16R wm. The resulting plasmid pGem-RG-B16R wm (5544 bp) was confirmed by DNA sequence analysis and directs the deletion of *B16R* gene from MVA-B ΔA41L genome.

### Construction of MVA-B ΔA41L/ΔB16R deletion mutant

We first generated the single deletion mutant MVA-B ΔA41L by screening for transient Red2/GFP co-expression [53] using dsRed2 and rsGFP genes as the transiently selectable markers. 3×10^6^ DF-1 cells were infected with MVA-B at a multiplicity of 0.05 PFU/cell and then transfected 1h later with 6µg of DNA from plasmid pGem-RG-A41L wm using Lipofectamine (Invitrogen, San Diego, CA) according to the manufacturer's recommendations. After 72h post-infection, the cells were harvested, lysed by freeze-thaw cycling, sonicated and used for recombinant virus screening. Deletion mutant was selected from progeny virus by 6 consecutive rounds of plaque purification in DF-1 cells and plaques were screened for Red2/GFP fluorescence. In the first two passages viruses from selected plaques expressed both fluorescent proteins, in the next two passages viral progeny from selected plaques expressed only one fluorescent marker (Red2 or GFP) and in the last two passages (six passages in total) viruses from selected plaques do not express any marker due to the loss of the fluorescent marker. MVA-B ΔA41L was obtained and the deletion of *A41L* gene was confirmed by PCR amplifying the *A41L* locus.

The double deletion mutant MVA-B ΔA41L/ΔB16R was constructed also by screening for transient Red2/GFP co-expression, following the same protocol detailed above. 3×10^6^ DF-1 cells were infected with MVA-B ΔA41L at a multiplicity of 0.05 PFU/cell and then transfected 1h later with 6µg of DNA from plasmid pGem-RG-B16R wm using Lipofectamine (Invitrogen, San Diego, CA). After 6 rounds of plaque purification MVA-B ΔA41L/ΔB16R was obtained and the deletion of *A41L* and *B16R* genes was confirmed by PCR amplifying the *A41L* and *B16R* locus.

The resulting MVA-B ΔA41L/ΔB16R virus was grown in CEF cells, purified by centrifugation through two 36% (w/v) sucrose cushions in 10mM Tris-HCl pH 9, and titrated in DF-1 cells by plaque immunostaining assay, using rabbit polyclonal antibody against vaccinia virus strain WR (Centro Nacional de Biotecnología; diluted 1∶1000) followed by anti-rabbit-HRP (Sigma; diluted 1∶1000). MVA-B ΔA41L/ΔB16R deletion mutant generated were free of contamination with mycoplasma (checked by specific PCR for mycoplasma) or bacteria (checked by growth in LB plates without ampicillin).

### PCR analysis of MVA-B ΔA41L/ΔB16R deletion mutant

To test the purity of MVA-B ΔA41L/ΔB16R deletion mutant, viral DNA was extracted from DF-1 cells mock-infected or infected at 2 PFU/cell with MVA, MVA-B, or MVA-B ΔA41L/ΔB16R. Primers RFA41L-AatII-F and LFA41L-BamHI-R (described above) spanning *A41L* flanking regions were used for PCR analysis of *A41L* locus. Primers LFB16R-AatII-F and RFB16R-BamHI-R (described above) spanning *B16R* flanking regions were used for PCR analysis of *B16R* locus. Both amplifications were made in a total volume of 25µl containing 0.3mM of each deoxynucleoside triphosphate, 0.3µM of each primer, 2mM MgCl_2_, 50 to 100ng of DNA template, reaction buffer (1×), and 1 unit of DNA polymerase Platinum *Taq* (Invitrogen). The PCR protocol consisted of an initial step of 5 min at 94°C, followed by 35 cycles of 1 min at 94°C, 1 min at 50°C, and 1∶30 min at 68°C. The final extension cycle was 7 min at 72°C. PCR products were resolved in 1% agarose gel in Tris-borate-EDTA (TBE) buffer with 0.5µg/ml ethidium bromide and were visualized using UV light. The deletions were also confirmed by DNA sequence analysis.

### Expression of HIV-1_BX08_ gp120 and HIV-1_IIIB_ Gag-Pol-Nef proteins by MVA-B ΔA41L/ΔB16R deletion mutant

To test the correct expression of HIV-1 proteins HIV-1_BX08_ gp120 and HIV-1_IIIB_ Gag-Pol-Nef (GPN), monolayers of DF-1 cells were mock-infected or infected at 2 PFU/cell with MVA, MVA-B or MVA-B ΔA41L/ΔB16R. At 24h post-infection, cells were lysed in Laemmli buffer, cells extracts fractionated by 12% SDS-PAGE and analyzed by Western blot using rabbit polyclonal anti-gp120 antibody against IIIB (Centro Nacional de Biotecnología; diluted 1∶3000) or polyclonal anti-gag p24 serum (ARP 432, NIBSC, Centralised Facility for AIDS reagent, UK; diluted 1∶3000) followed by anti-rabbit-HRP (Sigma; diluted 1∶5000) to evaluate the expression of gp120 and GPN proteins, respectively.

### Analysis of virus growth

To determine virus-growth profiles, monolayers of DF-1 cells grown in 12-well tissue culture plates were infected in duplicate at 0.01 PFU/cell with MVA-B or MVA-B ΔA41L/ΔB16R. Following virus adsorption for 60 min at 37°C, the inoculum was removed. The infected cells were washed once with DMEM without serum and incubated with fresh DMEM containing 2% FCS at 37°C in a 5% CO_2_ atmosphere. At different times post-infection (0, 24, 48 and 72 hours), cells were collected by scraping, freeze-thawed three times and briefly sonicated. The intracellular viruses were titrated by immunostaining as described above.

### Peptides

The HIV-1 peptide pools, with each purified peptide at 25µg per vial, were provided by the EuroVacc Foundation. They spanned the entire Env, Gag, Pol and Nef regions from clade B included in the virus vectors as consecutive 15-mers overlapped by 11 amino acids. The HIV-1_BX08_ gp120 protein (494 aa) was spanned by the Env-1 (aa: 1–251; 60 peptides) and Env-2 (aa: 241–494; 61 peptides) pools. The HIV-1_IIIB_ Gag-Pol-Nef fusion protein (1326 aa) was spanned by the following pools: Gag-1 (aa: 1–231; 55 peptides), Gag-2 (aa: 221–431; 50 peptides), GPN-1 (aa: 421–655; 56 peptides), GPN-2 (aa: 645–879; 56 peptides), GPN-3 (aa: 869–1103; 56 peptides) and GPN-4 (aa: 1093–1326; 56 peptides). For immunological analysis we grouped the peptides in three main pools: Env, Gag and GPN. The Env-pool comprises Env-1+Env-2; Gag-pool comprises Gag-1+Gag-2; and GPN-pool comprises GPN-1+GPN-2+GPN-3+GPN-4. HIV-1 peptide Gag-B (AMQMLKETI), from clade B, was produced at Centro Nacional de Biotecnología.

### Mice immunization schedule

BALB/c mice were purchased from Harlan. A DNA prime/MVA boost immunization protocol was performed as previously described [7]. Groups of animals (n = 8) received 100µg of DNA-B (50µg of pCMV-_BX08_gp120+50µg of pCDNA-_IIIB_GPN) by intramuscular route (i.m.) and two weeks later received an intraperitoneal (i.p.) inoculation of 1×10^7^ PFU of the corresponding recombinant vaccinia viruses (MVA-B or MVA-B ΔA41L/ΔB16R) in 200 µl of PBS. At 11 and 53 days after the last immunization 4 mice in each group were sacrificed and spleens processed for fresh IFN-γ ELISPOT and Intracellular Cytokine Staining (ICS) assays, to measure the adaptive and memory immune responses against HIV-1 antigens. Two independent experiments have been performed for the different groups.

### Fresh IFN-γ ELISPOT assay

Fresh IFN-γ ELISPOT assay was performed as previously described [54]. Briefly, 10^6^ and 5×10^5^ splenocytes (depleted of red blood cells) were plated in triplicate in 96-well nitrocellulose-bottomed plates previously coated with 6µg/ml of anti-mouse IFN-y mAb R4-6A2 (Pharmingen, San Diego, CA). HIV-1 peptide Gag-B from clade B was resuspended in RPMI 1640 supplemented with 10% FCS and added to the cells at a final concentration of 10µg/ml. Cells were incubated at 37°C, 5% CO_2_ for 48h, washed extensively with PBS containing 0.05% of Tween 20 (PBS-T) and incubated 2h at room temperature (RT) with a solution of 2µg/ml of biotinylated anti-mouse IFN-γ mAb XMG1.2 (Pharmingen, San Diego, CA) in PBS-T. Afterwards, plates were washed with PBS-T and 100µl of peroxidase-labeled avidin (Sigma, St. Louis, MO) at 1∶800 dilution in PBS-T was added to each well. After 1h of incubation at RT, wells were washed with PBS-T and PBS. The spots were developed by adding 1µg/ml of the substrate 3,3′-diaminobenzidine tetrahydrochloride (Sigma, St. Louis, MO) in 50mM Tris–HCl, pH 7.5 containing 0.015% hydrogen peroxide. The spots were counted with the aid of a stereomicroscope. Values are reported after the subtraction of background (cells with RPMI) and expressed as net spot-forming units per 10^6^ splenocytes.

### Intracellular Cytokine Staining assay (ICS)

The phenotypes of responding T cells were analyzed by ICS and fluorescence-activated cell sorting analysis as described elsewhere [3]. After an overnight rest, 5×10^6^ splenocytes (depleted of red blood cells) were resuspended in RPMI 1640 supplemented with 10% FCS and containing 1µl/ml Golgiplug (BD Biosciences) to inhibit cytokine secretion. Cells were seeded on M96 plates and stimulated with Gag-B peptide, Env-, Gag- or GPN-pools added to the cells at a final concentration of 5µg/ml. Cells were incubated at 37°C, 5% CO_2_, and then analyzed by ICS. After 6h of stimulation, cells were washed, stained for the surface markers, fixed, permeabilized using the BD Cytofix/Cytoperm™ Kit (Becton Dickinson) and stained intracellularly using the appropriate fluorochromes. To analyze the adaptive immune responses, the following fluorochrome-conjugated antibodies were used: CD3-FITC, CD4-Alexa 700, CD8-PerCP, IL-2-PE, IFN-γ-APC and TNF-α-PECY-7. For memory analyses, the following antibodies were used: CD4-Alexa 700, CD8-FITC, IFN-γ-PECY-7 and IL-2-Alexa-647. All antibodies were from BD Biosciences. Cells were acquired using an LSRII flow cytometer (Becton Dickinson) equipped with a high throughput system. The number of events ranged between 10^5^ and 10^6^. Dead cells were excluded using the violet LIVE/DEAD stain kit (Invitrogen). Lymphocytes were gated on a forward scatter area versus side scatter area pseudo-color dot plot. To analyze the adaptive immune responses, CD4^+^ and CD8^+^ events (gate previously on CD3^+^ cells) were gated versus IFN-γ, TNF-α and IL-2, and then combined together using the boolean operator. For memory analyses, CD4^+^ and CD8^+^ events were gated versus IFN-γ and IL-2, and then combined together using the boolean operator. Sample analysis was performed using FlowJo version 8.5.3 (Tree Star, Ashland, OR).

### Antibody measurements by Enzyme-linked Immunosorbent Assay (ELISA)

Antibodies anti-HIV-1 gp160LAV envelope protein were measured by ELISA as previously described [7]. Briefly, high binding polystyrene microtitre plates (Nunc) were coated with 100µl of the purified HIV-1 gp160LAV envelope protein (Protein Sciences) diluted at 2µg/ml in 0.05M carbonate–bicarbonate buffer pH 9.6 overnight at 4°C. The wells were washed twice with PBS-T and blocked with PBS containing 10% FCS (blocking solution) during 1h at 37°C. Serum samples diluted 1/100 in blocking solution were added in a volume of 100µl/well and incubated 2h at 37°C. Plates were washed three times with PBS-T before the detection antibody was added. Peroxidase-conjugated goat anti-mouse immunoglobulin G (IgG) antibody (Southern Biotechnology Associated, Birmingham, Ala) was diluted 1∶1000 in PBS-T and incubated for 1h at 37°C. The plates were washed again three times with PBS-T and 3,3′,5,5′ Tetramethylbenzidine (TMB) (Sigma) was used to reveal the reaction. After 10–15 min of incubation at RT, the reaction was stopped by adding 2N H_2_SO_4_, and absorbance was measured at 450nm on a Multiskan Plus plate reader (Labsystem, Chicago, Ill).

### Statistical procedures

For the statistical analysis, we have developed a novel approach that corrects measurements for the medium response (RPMI) and at the same time it allows the exact calculation of confidence intervals and p-values of hypothesis tests. For ELISPOT and ICS statistical analysis, it was proceeded as previously described [55]. Given the total number of cells, 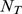, and the number of cells responding to a given antigen, 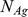, an estimate of the proportion of cells responding to this antigen is given by 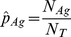. The Bayesian *a posteriori* distribution of 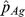 without any *a priori* assumption (i.e., assuming that the true proportion is uniformly distributed between 0 and 1) is the Beta distribution with parameters 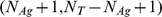 [56]. Let us call 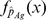 the corresponding probability density function *a posteriori*. Analogously, we can derive the distribution of the proportion of cells responding to RPMI, obtaining the distribution 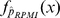. To test whether the antigen response is significantly larger than the RPMI response, we computed the probability density function of the variable 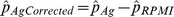 as 
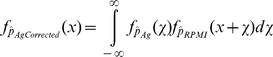
. The cumulative density function of this variable is defined in the usual way 
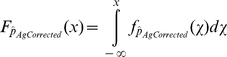
. The 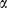 percentile of this variable is defined as 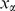 such that 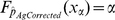. We computed the symmetric 95% confidence interval for the RPMI corrected proportion as 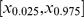. Finally, we consider 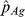 to be significantly larger than 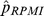 if 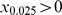. In such a case, 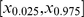 gives the 95% symmetric confidence interval for 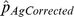. The average 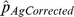 is computed as the expected value of 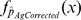 (note that this expected value needs not be in the middle of the confidence interval, 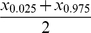). Whenever two corrected proportions need to be summed, 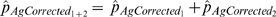, we convolved their probability density functions to obtain the probability density function of the summed proportion, 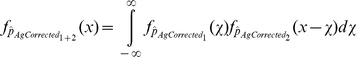, in this way confidence intervals for any sum of corrected proportions can be obtained. Antigen responses were not added unless each component was significantly larger than the corresponding RPMI.

In the ELISPOT experiment, three replicates were obtained for each kind of antigen. The average response to that antigen was computed using only the corrected proportions significantly larger than the corresponding RPMI. The division implied by the averaging process needed a reinterpolation of the probability density function which we carried out using cubic splines as implemented in MATLAB 2008a.

## Supporting Information

Table S1 Comparative HIV-1-specific immune responses elicited in different animal models and humans using DNA prime/poxvirus boost (MVA or NYVAC) immunization protocols from the EuroVacc trials. The induction of HIV-1-specific immune responses measure by ELISPOT and ICS after boost with different poxvirus vectors (MVA or NYVAC) in different animal models (mice or non-human primates) or humans (Phase I clinical trials) is indicated. The different DNA and recombinant poxvirus used in the prime/boost contains different HIV-1 genes (Env, Gag, Pol and Nef) from different clades (B or C), and are indicated in the corresponding row. HIV-1-specific IFN-γ secreting cells are measured by ELISPOT, and the magnitude and breath induced are indicated. HIV-1-specific CD4+ and CD8+ T cells are measured by ICS, and the magnitude, breath and polyfuncionality induced are indicated. ND, not determine.
(0.08 MB DOC)
