# Supplementary material for: Immunogenic Profiling in Mice of a HIV/AIDS Vaccine Candidate (MVA-B) Expressing Four HIV-1 Antigens and Potentiation by Specific Gene Deletions
Source: PLoS One. 2010 Aug 24;5(8):e12395. doi: 10.1371/journal.pone.0012395 (PMC2927552; doi:10.1371/journal.pone.0012395)
Supplement: Table S1 — Comparative HIV-1-specific immune responses elicited in different animal models and humans using DNA prime/poxvirus boost (MVA or NYVAC) immunization protocols from the EuroVacc trials. The induction of HIV-1-specific immune responses measure by ELISPOT and ICS after boost with different poxvirus vectors (MVA or NYVAC) in different animal models (mice or non-human primates) or humans (Phase I clinical trials) is indicated. The different DNA and recombinant poxvirus used in the prime/boost contains different HIV-1 genes (Env, Gag, Pol and Nef) from different clades (B or C), and are indicated in the corresponding row. HIV-1-specific IFN-γ secreting cells are measured by ELISPOT, and the magnitude and breath induced are indicated. HIV-1-specific CD4+ and CD8+ T cells are measured by ICS, and the magnitude, breath and polyfuncionality induced are indicated. ND, not determine. (0.08 MB DOC) [file pone.0012395.s001.doc]

| EuroVacc trials (preclinical and clinical) | | | | | | |
| --- | --- | --- | --- | --- | --- | --- |
| Animal model | HIV-1 immune responses | | | | DNA prime/poxvirus boost (MVA or NYVAC)  [Immunogens] | References |
|  | ELISPOT |  | ICS | |  |  |
|  | IFN-γ |  | CD4^+^ T-cell responses | CD8^+^ T-cell responses |  |  |
| Mouse | - Magnitude: High  - Breath: Env>GPN >Gag |  | - ND | - ND | DNA-B/MVA-B  [Env (gp120) and Gag-Pol-Nef (GPN) from subtype B] | [7,9] |
|  | - ND |  | - Magnitude: Low  - Breath: Env>GPN >Gag  - Polyfunctionality: High | - Magnitude: High  - Breath: Env>GPN >Gag  - Polyfunctionality: High | DNA-B/MVA-B  [Env (gp120) and Gag-Pol-Nef (GPN) from subtype B] | García-Arriaza et al. (this manuscript) |
| Non-human primate | - Magnitude: High  - Breath: Env>Gag>Pol>Nef |  | - Magnitude: High  - Breath: Env>Gag  - Polyfunctionality: High | - Magnitude: High  - Breath: Env>Gag  - Polyfunctionality: High | DNA/MVA  [SHIV_89.6P_ Env (gp120) and SIV_mac239_ Gag-Pol-Nef] | [3] |
| Mouse | - Magnitude: High  - Breath: Env>GPN >Gag |  | - ND | - ND | DNA-B/NYVAC-B  [Env (gp120) and Gag-Pol-Nef (GPN) from subtype B] | [7,9] |
|  | - Magnitude: High  - Breath: Env>GPN >Gag |  | - Magnitude: High  - Breath: Env>GPN>Gag  - Polyfunctionality: High | - Magnitude: Low  - Breath: Env>GPN>Gag  - Polyfunctionality: High | DNA-B/NYVAC-B  [Env (gp120) and Gag-Pol-Nef (GPN) from subtype B] | [55] |
| Non-human primate | - Magnitude: High  - Breath: Env>Gag>Pol>Nef |  | - Magnitude: High  - Breath: Env>Gag  - Polyfunctionality: High | - Magnitude: Low  - Breath: Env>Gag  - Polyfunctionality: High | DNA/NYVAC  [SHIV_89.6P_ Env (gp120) and SIV_mac239_ Gag-Pol-Nef] | [3] |
| Mouse | - Magnitude: High  - Breath: Env>GPN >Gag |  | - ND | - ND | DNA-C/NYVAC-C  [Env (gp120) and Gag-Pol-Nef (GPN) from subtype C] | [8] |
|  | - ND |  | - ND | - Magnitude: High  - Breath: Env>Pol  - Polyfunctionality: ND | DNA-C/NYVAC-C  [Env (gp120) and Gag-Pol-Nef (GPN) from subtype C] | [49] |
| Non-human primate | - Magnitude: High  - Breath: Env>Pol >Gag>Nef |  | - Magnitude: High  - Breath: Env>Pol >Gag>Nef  - Polyfunctionality: High | - Magnitude: High  - Breath: Env>Pol >Gag>Nef  - Polyfunctionality: High | DNA-C/NYVAC-C  [Env (gp120) and Gag-Pol-Nef (GPN) from subtype C] | [50] |
| Human | - Magnitude: High  - Breath: Env>Pol >Gag>Nef |  | - Magnitude: High  - Breath: Env>GPN  - Polyfunctionality: High | - Magnitude: Low  - Breath: Env>GPN  - Polyfunctionality: High | DNA-C/NYVAC-C  [Env (gp120) and Gag-Pol-Nef (GPN) from subtype C] | [10,51] |
|  | - Magnitude: High  - Breath: Env>Pol >Gag>Nef |  | - Magnitude: High  - Breath: Env>GPN  - Polyfunctionality: High | - Magnitude: Low  - Breath: Env>GPN  - Polyfunctionality: High | DNA-C/NYVAC-C  [Env (gp120) and Gag-Pol-Nef (GPN) from subtype C] | [50] |

**Table S1. Comparative HIV-1-specific immune responses elicited in different animal models and humans using DNA prime/poxvirus boost (MVA or NYVAC) immunization protocols from the EuroVacc trials.** The induction of HIV-1-specific immune responses measure by ELISPOT and ICS after boost with different poxvirus vectors (MVA or NYVAC) in different animal models (mice or non-human primates) or humans (Phase I clinical trials) is indicated. The different DNA and recombinant poxvirus used in the prime/boost contains different HIV-1 genes (Env, Gag, Pol and Nef) from different clades (B or C), and are indicated in the corresponding row. HIV-1-specific IFN-γ secreting cells are measured by ELISPOT, and the magnitude and breath induced are indicated. HIV-1-specific CD4^+^ and CD8^+^ T cells are measured by ICS, and the magnitude, breath and polyfuncionality induced are indicated. ND, not determine.
